# Alzheimer’s disease and architectural topologies of residential care facilities

**DOI:** 10.1192/j.eurpsy.2026.10478

**Published:** 2026-07-31

**Authors:** N. Bouayed Abdelmoula, E. Abdelmoula, B. Abdelmoula

**Affiliations:** 1 LR23ES07, Genomics of Signalopathies at the service of Precision Medicine, Medical University of Sfax, Sfax; 2 LR21ES19 UMRAN, National School of Architecture and Urbanism of Tunis; 3 3Doctoral school of architectural sciences and engineering (ED-SIA), Carthage University, Carthage, Tunisia

## Abstract

**Introduction:**

It is now well established that the architectural design of residential care facilities for people with cognitive decline must meet both the specific needs of patients and the requirements of the nursing staff. For patients with Alzheimer’s disease (AD), a new residential typology has emerged over the past 40 years, based on person-centered care and shaped by advances in neuroscience, gerontology and environmental psychology.

**Objectives:**

The objective of this study is to identify potential recommendations and reference models in architectural design for residential care facilities, with a particular focus on environments adapted to the needs of people with AD.

**Methods:**

We conducted a comprehensive review of the scientific literature using the following keywords: Alzheimer’s disease and architecture design.

**Results:**

Our analysis confirms that residential environments designed specifically for people with AD must go far beyond mere functionality and aesthetics. We identified eight fundamental architectural features that, combined, improve the behavior, emotional well-being, and cognitive abilities of people with AD: small scale, simple domestic units, clear spatial organization, adapted sensory stimulation, safety, accessibility, environmental comfort (lighting and temperature), and technological integration. These architectural features align with international recommendations for dementia- and AD-friendly architecture. Guidelines from organizations such as the Alzheimer’s Association (United States) and the Dementia Services Development Centre (DSDC) at the University of Stirling (Scotland) converge towards a typology based on: small-scale units that facilitate orientation and create a familial atmosphere; simple and secure circulation paths with looped routes and minimal decision points; sensory spaces and access to nature to stimulate the senses and promote emotional well-being; integration of safety measures without stigma, ensuring autonomy while preventing risks; spaces dedicated to social interaction and participation in daily activities; and the adaptation of environments to meet the needs of caregivers and families, fostering a comprehensive support system. In addition, the WHO advocates for “dementia-friendly” environments focused on maintaining autonomy, spatial orientation, and social integration, which corresponds closely to our typology centered on the cognitive and emotional needs of people with AD. Innovative models such as the Green House Project in the United States exemplify the effectiveness of organizing spaces as “houses within a house,” promoting a familiar and warm atmosphere.

**Conclusions:**

Designing residential environments for this population demands a holistic, multidisciplinary approach that prioritizes cognitive, emotional, and sensory needs, positioning architecture not necessarily as a container but as a therapeutic tool to enhance autonomy, well-being, and quality of life.

**Disclosure of Interest:**

None Declared

